# Non-syndromic retinal dystrophy associated with biallelic variation of *SUMF1* and reduced leukocyte sulfatase activity

**DOI:** 10.1111/cge.14573

**Published:** 2024-06-11

**Authors:** Siying Lin, Anthony G Robson, Dorothy A Thompson, Karolina M Stepien, Robin Lachmann, Emma Footitt, Ola Czyz, Shwetha Chandrasekhar, Elena Schiff, Christos Iosifidis, Graeme C Black, Michel Michaelides, Omar A Mahroo, Gavin Arno, Andrew R Webster

**Affiliations:** 1 NIHR Biomedical Research Centre at Moorfields Eye Hospital and the UCL Institute of Ophthalmology, UK; 2 UCL Institute of Ophthalmology, University College London, UK; 3 Tony Kriss Visual Electrophysiology Unit, Department of Clinical and Academic, Department of Ophthalmology, Sight and Sound Centre, Great Ormond Street Hospital for Children , UK; 4 UCL Great Ormond Street Institute of Child Health, University College London, UK; 5 Adult Inherited Metabolic Disorders, Salford Royal Organisation, Northern Care Alliance NHS Foundation Trust, UK; 6 Charles Dent Metabolic Unit, National Hospital for Neurology and Neurosurgery, UK; 7 Department of Metabolic Paediatrics, Great Ormond Street Hospital, UK; 8 Manchester Royal Eye Hospital, Manchester University NHS Foundation Trust, UK; 9 Department of Ophthalmology, St Thomas’ Hospital, UK; 10 Greenwood Genetic Center, South Carolina, USA

**Keywords:** multiple sulfatase deficiency, retinal dystrophies, MSD, SUMF1, lysosomal storage disorder

## Abstract

Biallelic variants in *SUMF1* are associated with multiple sulfatase deficiency (MSD), a rare lysosomal storage disorder typically diagnosed in early infancy or childhood, marked by severe neurodegeneration and early mortality. We present clinical and molecular characterisation of three unrelated patients aged 13 to 58 years with milder clinical manifestations due to *SUMF1* disease variants, including two adult patients presenting with apparent non-syndromic retinal dystrophy. Whole genome sequencing identified biallelic *SUMF1* variants in all three patients; patient 1 homozygous for a complex allele c.[290G>T;293T>A]; p.[(Gly97Val);Val98Glu)], patient 2 homozygous for c.866A>G; p.(Tyr289Cys), and patient 3 compound heterozygous for c.726-1G>C and p.(Tyr289Cys). Electroretinography indicated a rod-cone dystrophy with additional possible inner retinal dysfunction in all three patients. Biochemical studies confirmed reduced, but not absent, sulfatase enzyme activity in the absence of extra-ocular disease (patient 1) or only mild systemic disease (patients 2,3). These cases are suggestive that non-null *SUMF1* genotypes can cause an attenuated clinical phenotype, including retinal dystrophy without systemic complications, in adulthood.

## Introduction

The *SUMF1* (Sulfatase-Modifying Factor 1) gene encodes for the formylglycine-generating enzyme (FGE), which plays a vital role in post-translational modification and catalytic activation of all 17 cellular sulfatase enzymes in humans. These enzymes are essential for multiple cellular functions including hormone regulation, cellular degradation, and modulation of signalling pathways (1). FGE converts a highly-conserved cysteine residue within the catalytic domain of all sulfatase enzymes into Cα-formylglycine, a step necessary for sulfatase enzyme activation (1,2).

Biallelic variants in *SUMF1* lead to multiple sulfatase deficiency (MSD, MIM #272200), an extremely rare autosomal recessive multisystem lysosomal storage disorder (LSD), with only approximately 150 affected individuals reported to date (3). In MSD, impairment of FGE function leads to insufficient activation of all sulfatases to varying degrees, with each deficiency contributing to specific clinical manifestations. Ocular features are present in 50-80% of patients with MSD, and include corneal clouding, optic atrophy and retinal dystrophy (3).

This study presents a clinical, biochemical and molecular characterisation of three patients with an attenuated MSD disease phenotype resulting from biallelic *SUMF1* variants, including an adult individual with non-syndromic retinal dystrophy, representing the mildest phenotypes associated with *SUMF1* gene dysfunction reported to date.

## Materials and methods

This study adhered to the tenets of the Declaration of Helsinki and received relevant local research ethics approval. Written informed consent was obtained from all patients and their relatives participating. A comprehensive clinical history was obtained, and in-depth phenotyping, including ophthalmic, systemic and biochemical assessments, was conducted where possible. Whole genome sequencing (WGS) was performed for all three patients. Further details on electrophysiology, biochemical assessments, and genetic testing can be found in the supplemental information.

## Results

### Clinical presentation

Patient 1 (GC15080) is a 37-year-old Asian Pakistani male, who first noticed a reduction in central vision at the age of 13 years, and was diagnosed with a retinal dystrophy aged 15 years. He suffers from from occasional backache and sciatica but is otherwise fit and well. Parental consanguinity was noted, but there is no family history of vision problems.

Patient 2 (GC19954) is a 59-year-old British Caucasian female diagnosed with retinal dystrophy in her early 40s after experiencing difficulty with reading. She also has glaucoma and Fuch’s corneal endothelial dystrophy. Her medical history includes a deep vein thrombosis and a transient ischaemic attack diagnosed at age 47 and 54 years respectively.

Patient 3 is a 13-year-old British Caucasian male with a complex medical history (see Table 1). His mother and three older siblings have all been diagnosed with Ehlers-Danlos syndrome, and two older siblings have also been diagnosed with autism. While visual symptoms were not initially apparent, the patient reported problems with night and peripheral vision on direct inquiry.

**Figure 1:**
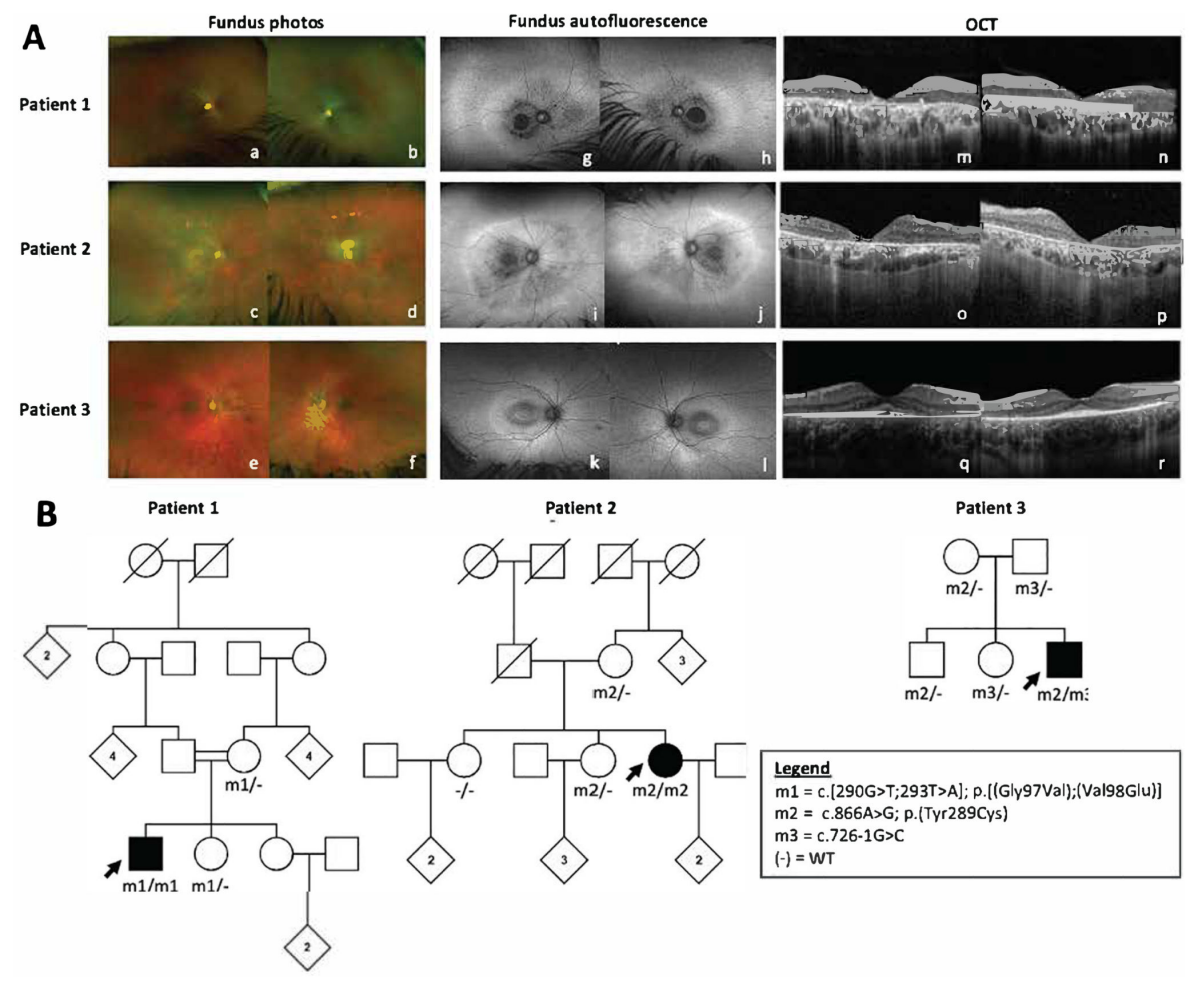
Clinical findings and family pedigrees for patients 1-3. (A) Optos pseudocolour fundus photographs (a-f), Optos fundus autofluorescence (g-l) and Spectralis optical coherence tomography (OCT) images (m-r) for patients 1-3 with biallelic *SUMF1* variants and retinal dystrophy (B) Family pedigrees showing genotypes and familial segregation where available.

All three patients had a symmetrical retinal dystrophy with macular involvement on ophthalmological examination (Figure 1A). The electroretinogram (ERG) in patients 1 and 2 was indicative of a rod-cone dystrophy with additional inner retinal dysfunction, and with severe macular involvement. The ERG in patient 3 was consistent with severe and selective rod system dysfunction (Supplemental Information).

A summary of ocular and systemic findings for all three patients is provided in Table 1.

### Genetic results

For patients 1 and 2, WGS and subsequent virtual gene panel analysis excluded pathogenic genotypes in known inherited retinal disease (IRD) genes. Further analysis focused on prioritising rare protein-altering variants with a population frequency of <0.01 in control databases (Genome Aggregation Database; gnomAD v4.0.0, and 100kGP), and the only plausible candidate disease variants identified were located within the *SUMF1* gene. Patient

1 was homozygous for a complex allele (NM_182760) c.[290G>T;293T>A]; p.[(Gly97Val);(Val98Glu)], whilst patient 2 was homozygous for missense variant c.866A>G; p.(Tyr289Cys).

WGS in patient 3 identified compound heterozygosity for *SUMF1* c.726-1G>C and c.866A>G; p.(Tyr289Cys).

Segregation analysis in all three patients is consistent with the variants being inherited in *trans* (Figure 1B)

Table 2 provides an overview of population frequency and *in silico* predictions for all *SUMF1* variants reported in this study, none of which have been previously reported in the literature.

Due to markedly low leucocyte arylsulfatase A (ASA) levels in all three patients, the genomic data was scrutinized for the ASA pseudodeficiency allele *ARSA* (NM_001085427) c.[1055C>G; *96A>G]; p.[(Asn352Ser);(?)]. Patient 1 was heterozygous for this allele, whilst patients 2 and 3 did not carry this allele.

## Discussion

This study describes three individuals with an attenuated systemic phenotype, including non-syndromic retinal dystrophy, due to biallelic variants in *SUMF1. SUMF1* variants are typically associated with MSD, marked by severe progressive neurological deterioration and early mortality (4).

In recent years, there is growing recognition that genes initially associated with severe LSDs, such as neuronal ceroid lipofuscinosis (*CLN3* and *MFSD8*) and mucopolysaccharidosis type IIIC (*HGSNAT*), can also cause non-syndromic retinal dystrophy (5–7). For individual 1 reported in this study, comprehensive clinical evaluations at age 37 years have not identified any extraocular features associated with MSD. This individual with non-syndromic retinitis pigmentosa therefore represents the mildest *SUMF1*-associated phenotype to date.

*SUMF1* variants are typically associated with a variable disease spectrum determined by the severity of FGE protein instability and its residual catalytic ability (4,8). Most *SUMF1* variants identified in individuals with MSD are missense variants that likely permit residual enzyme activity, and it has been noted that all MSD patients have reduced but measurable levels of sulfatase activity (9). It is therefore believed that MSD is largely caused by hypomorphic *SUMF1* variants; indeed, biallelic loss of function alleles in *SUMF1* have only very rarely been reported in cases of hydrops fetalis, and appear incompatible with life (10,11).

Clinical and biochemical analyses support our hypothesis that the novel missense p.(Tyr289Cys) and p.[(Gly97Val);(Val98Glu)] alleles identified in this study are particularly mild alleles, resulting in a limited ocular phenotype (p.[(Gly97Val);(Val98Glu)]; patient 1) or an attenuated MSD phenotype (p.(Tyr289Cys); patients 2 and 3). The biochemical evidence shows reduced enzyme activity across several sulfatases in all three patients, although these levels are not as markedly low as typically seen in classic MSD presentations. Notably, the p.(Tyr289Cys) variant exhibits significant differences in predicted effect according to Revel and AlphaMissense scores; Revel predicts pathogenicity, whilst AlphaMissense suggests it is likely benign. AlphaMissense uniquely incorporates AlphaFold data, which is not included in the 13 tools used to calculate the Revel ensembl score, highlighting the potential for AlphaMissense to provide a more accurate reflection of the variant’s moderate effect. Further detailed structural and biochemical studies investigating the impact of these *SUMF1* variants on FGE subcellular localisation, intracellular retention, enzymatic activity, and protein stability would be highly beneficial in understanding the molecular mechanisms through which these hypomorphic variants contribute to milder clinical presentations (12).

*Sumf1* knock-out mice have complete deficiency of sulfatase enzyme activity, and exhibit a high mortality rate in the first weeks of life (13). Hypomorphic *Sumf1* mice instead show a predominant retinal phenotype with a reduction in rod and cone a- and b-wave on ERG testing, and a reduction in retinal outer nuclear layer thickness consistent with rod and cone degeneration (14). Retinal dystrophies have been described in patients with single sulfatase deficiencies including MPS type II (*IDS*), III-A (*SGSH*), III-D (*GNS*), IV-A (*GALNS*), X (*ARSK*) as well as Usher syndrome type IV (*ARSG*) (15–20), and the retinopathy identified in patients 1-3 are likely due to the resulting deficiencies in one or more of these sulfatases. Within the retina, it is thought that impairment of sulfatase enzyme activity leads to the progressive accumulation of glycosaminoglycans in the retinal pigment epithelial cells as well as the inter-photoreceptor matrix, leading to progressive photoreceptor loss (21). However, the precise pathophysiological mechanisms underlying the selective retinal involvement remains unclear.

These cases reveal the existence of attenuated forms of MSD, expanding the phenotypic spectrum associated with *SUMF1* variants, and highlighting a novel association with non-syndromic retinal dystrophy. The retinal phenotype is characterised by posterior pole autofluorescence changes and outer retinal disruption in the macular region, with ERGs consistent with either a rod-cone dystrophy with evidence of additional inner retinal dysfunction, or severe rod system dysfunction.

Classical forms of LSD typically exhibit significantly reduced enzyme activity, while attenuated forms as observed in our case series may retain residual or near-normal levels, limiting the diagnostic utility of biochemical tests. Recognising that attenuated forms of LSD may present with milder symptoms and atypical blood or imaging findings is essential. *SUMF1* and other LSD genes linked to retinal phenotypes should be incorporated into IRD gene panels for accurate molecular diagnoses, enabling timely referral to metabolic specialists for comprehensive evaluations, management of systemic associations, and facilitating access to promising therapeutic approaches that could improve patient outcomes (22).

## Supplementary Material

Supplemental data

## Figures and Tables

**Table 1:** Clinical and biochemical studies for patients 1-3

	Patient 1 (GC15080)	Patient 2 (GC19954)	Patient 3
Sex, ethnicity	Male, Asian Pakistani	Female, Caucasian	Male, Caucasian
*SUMF1* genotype	c. 290G>T; p.(Gly97Val) c.293T>A ; p.(Val98Glu) Homozygous for both variants	c.866A>G ; p.(Tyr289Cys) Homozygous	c.866A>G ; p.(Tyr289Cys) c.726-1G>C Compound heterozygous
Presenting symptom (age, y)	Reduced central vision (13)	Reduced reading vision (40s)	Multisystem (childhood)
Age at last examination (y)	37	58	13
**Ocular features**			
BCVA (Snellen)	HM both eyes	3/60 both eyes	6/7.5 both eyes
Cornea	Clear	Endothelial guttata	Clear
Lens	Clear	Mild cataracts	Clear
Fundus	Central macular atrophy	Mild pigmentary changes at the central macula	Mild RPE mottling at posterior pole
Auto-fluorescence	Posterior pole hyper autofluorescence with central mottled hypo autofluorescence	Posterior pole hyper autofluorescence with central mottled hypo autofluorescence	Narrow perimacularring of hyperautofluorescence
OCT	Outer retinal disruption	Outer retinal disruption	Perifoveal loss of outer segment structure with a “bulls-ey” appearance
ERG	Severe generalised rod photoreceptor dysfunction with mild cone system involvement and severe macular involvement. **Reduced DA3 & DA10 ERG b:a ratios (additional inner retinal rod system dysfunction)**	Severe generalised rod/cone photoreceptor dysfunction with severe macular involvement. **Reduced DA3, DA10 and LA3 ERG b:a ratios and (additional inner retinal dysfunction)**	LA ERGs normal. DA10 ERG a-wave borderline. **Reduced DA3 & DA10 ERG b:a ratios (inner retinal rod system dysfunction)**
Other	-	Glaucoma, FCED	-
**Non-ocular features**			
Cardiac evaluation	Not available	Echocardiography: LVH with normal systolic function, grade 1 diastolic dysfunction	Normal echocardiography
Abdominal evaluation	No organomegaly	Mild fatty liver and gallstones	Small kidneys, mildly enlarged liver with no intrahepatic lesions
Skeletal survey	No dysostosis multiplex	No dysostosis multiplex	Scoliosis and subtle thoracolumbar kyphosis. No dysostosis multiplex
Neuroimaging	No leukodystrophy, incidental pituitary microadenoma under investigation	Small vessel disease	Mild prominence of perivascular spaces and mild ventriculomegaly involving the lateral ventricles. No emergent features of MSD
Other features	-	-	Autism, oral aversion and feeding difficulties, short stature, recurrent ear and throat infections, mild OSA, hypermobility and POTS (EDS type 3)
**Biochemical testing**			
Arylsulphatase A	↓ ↓	↓ ↓	↓ ↓
Heparin Sulphamidase	↓	↓	↓ ↓
Iduronate Sulphatase	↓	Normal	Normal
Galactose-6-sulphatase	Normal	Normal	Normal
N-acetyl glucosamine-6-sulphatase	Not available	Not available	↓ ↓
Urinary glycosaminoglycan	Presence of chondroitin sulphate, heparan sulphate and trace dermatan sulphate	Normal	Presence of chondroitin sulphate, heparan sulphate and dermatan sulphate

Abbreviations: BCVA, best corrected visual acuity; DA, dark-adapted; ECG, electrocardiogram; EDS, Ehlers-Danlos syndrome; ERG, electroretinogram; FCED, Fuchs corneal endothelial dystrophy; LA, light-adapted; LVH, left ventricular hypertrophy; MSD, multiple sulfatase deficiency; OCT, optical coherence tomography, OSA, obstructive sleep apnoea, POTS, postural orthostatic hypotension syndrome, RPE, retinal pigment epithelium; y, year; ↓, reduced ; ↓↓, significantly reduced

**Table 2:** *SUMF1* genetic data for patients 1-3

Patient	Ethnicity	Variant (nucleotide)	Variant (protein)	Genotype	gnomAD MAF	*In silico* predictions †			ClinVar (ID)
						Revel	AlphaMissense	SpliceAI	
1(GC15080)	Asian Pakistani	c. 290G>T	p.(Gly97Val)	Hom	Absent	Pathogenic (0.928)	Likely pathogenic (0.8002)	Benign (0.01)	Absent
		c.293T>A	p.(Val98Glu)	Hom	Absent	Uncertain (0.53)	Likely benign (0.1167)	Benign (0)	Absent
2 (GC19954)	Caucasian	c.866A>G	p.(Tyr289Cys)	Hom	0.00001847	Pathogenic (0.862)	Likely benign (0.299)	Benign (0)	Uncertain significance (1306425)
3	Caucasian	c.866A>G	p.(Tyr289Cys)	Het					
		c.726-1G>C	-	Het	0.000003601	NA	NA	Splice-altering (0.97)	Likely pathogenic (1067238)

† REVEL is an ensemble score based on 13 individual scores for predicting the pathogenicity of missense variants. AlphaMissense scores can be interpreted as the approximate probability of a variant being clinically pathogenic. SpliceAI delta scores can be interpreted as the probability that the variant affects splicing at any position within a +/-500bp window around it. Scores for REVEL, AlphaMissense and SpliceAl range from 0 to 1, with higher scores indicating a higher probability of the variant being damaging or having a splice altering effect. Abbreviations: gnomAD, Genome Aggregation Database v4.0.0; Het, heterozygous, Hom, homozygous; MAF, minor allele frequency

## Data Availability

Further details of the genome sequencing data presented in the study are available via direct contact with the corresponding author.
