# Supplementary material for: Non-syndromic retinal dystrophy associated with biallelic variation of SUMF1 and reduced leukocyte sulfatase activity
Source: Clin Genet. Author manuscript; Available in PMC 2024 Oct 1. (PMC7616411; doi:10.1111/cge.14573)
Supplement: Supplemental data [file EMS196839-supplement-Supplemental_data.docx]

**SUPPLEMENTAL INFORMATION**

**1. Visual electrophysiology**

**Methods**

Visual electrophysiology included dark-adapted (DA) and light-adapted (LA) full-field electroretinogram (ERG) and pattern electroretinogram (PERG) testing, incorporating the International Society for Clinical Electrophysiology of Vision (ISCEV) standards (1,2). Dark-adapted red flash ERGs were additionally recorded (3).

**Results**

Patient 1: Full-field ERG (Supplemental Figure S1), performed at the age of 15 years, revealed an undetectable scotopic dim flash (DA 0.01) ERG, a severely abnormal DA red flash ERG b-wave, a strong flash (DA 10) ERG that showed marked a-wave reduction and LA ERGs of normal timing and borderline amplitude. The DA 10 ERG also had a reduced b:a amplitude ratio (an electronegative ERG), likely partly attributable to dark-adapted cone system contributions (see Discussion). The PERG P50 component was undetectable, in keeping with severe macular cone involvement (4). Serial ERG recordings indicated mild progressive rod and cone dysfunction up to the age of 20 years with less progression over the following decade (Supplemental Figure S3).

Patient 2: Full-field ERGs, performed at age 48 years, were consistent with a relatively mild rod-cone dystrophy with PERG P50 evidence of severe macular involvement. Additionally, DA 3, DA 10 ERGs and the LA 3 ERG had a subnormal b:a amplitude ratio, in keeping with mild dysfunction post-phototransduction.

Patient 3: Paediatric skin flash ERGs (5) revealed attenuated rod-driven b-waves and a marked electronegative strong flash scotopic ERG with an a-wave index of photoreceptor function at the 5^th^ centile. A subsequent ISCEV standard full-field ERG confirmed profoundly electronegative strong flash dark-adapted ERGs, with a-wave amplitude at the lower reference limits. Although oscillatory potentials (OP) were preserved, the op2 was smaller than typical. Light-adapted full-field ERG peak times were within reference range but amplitudes fell at the lower reference limit for amplitude (Supplemental Figure S2). Pattern reversal visual evoked potentials were preserved indicating indirectly some preservation of the macular and foveal pathways to the striate cortex. In view of the ERG phenotype and the patients restricted diet, vitamin A levels were investigated, and were reported as normal.

**Discussion**

A shared and distinctive ERG feature among all three patients is the consistently reduced b:a amplitude ratio in their DA strong flash ERGs. Selective b-wave impairment and/or electronegative ERGs (where the a-wave larger than the b-wave) have also been noted in other systemic metabolic disorders, such as congenital disorder of glycosylation due to phosphomannomutase deficiency (*PMM2*) (6,7), mucopolysaccharidosis type 1 (*IDUA*) (8), and neuronal ceroid lipofuscinosis type 3 (*CLN3*) (9), and suggest additional inner retinal dysfunction post-phototransduction. It is also worth noting that these findings may relate to dark-adapted cone system contributions, exposed in the near-absence of rod function, which has been described in other retinal disorders associated with severe or selective rod photoreceptor dysfunction (10–14). Further ERG examinations in additional cases of *SUMF1*-associated retinal dystrophy will help establish whether a reduced b:a ratio is a consistent and distinguishing phenotypic feature of the condition.

**Supplemental Figure S1**


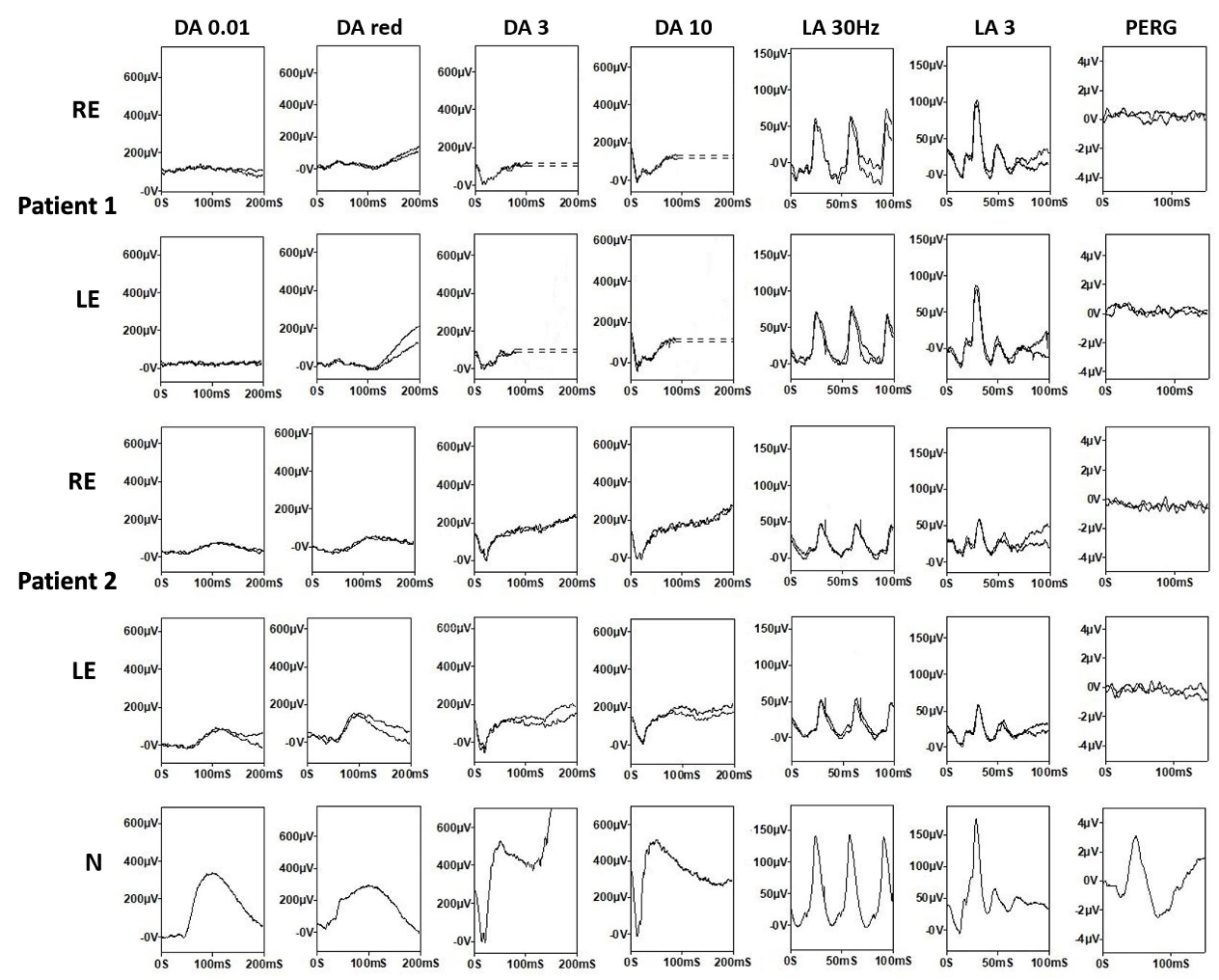


Full-field ERGs (DA 0.01, DA red, DA 3, DA 10, LA 30Hz, and LA 3) and PERG from the right eye (RE) and left eye (LE) of patient 1 (rows 1 and 2) and patient 2 (rows 3 and 4) and from a representative unaffected control subject (N; row 5). All patient traces are superimposed to demonstrate reproducibility. Broken lines after the ERG b-waves replace blink artefacts for clarity.

In patient 1 the DA 0.01 ERG and rod system mediated b-wave of the DA red flash ERG are undetectable and the DA 3 and DA 10 ERG a-waves markedly subnormal with additional reduction in the b:a ratio. The LA 30Hz ERG and LA 3 ERGs are of normal timing and are of borderline amplitude. In patient 2 the DA ERGs are subnormal, including DA 3 and DA 10 a-wave reductions with low b:a ratios; LA 30 ERGs show marginal delay and LA 3 ERG show mild reduction in the b:a ratio. PERGs are undetectable in both patients, in keeping with severe macular dysfunction.

**Supplemental Figure S2**


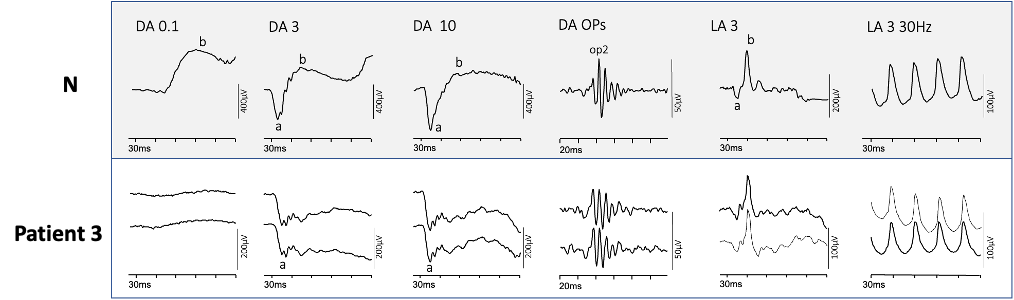


Typical full-field ERGs are shown to ISCEV standard stimuli under dark DA and light LA adapted in the upper shaded panel (N). The lower panel shows full-field ERGs from patient 3, aged 9 years. The DA 0.01 rod driven b-wave is markedly attenuated. Mixed rod cone DA 3 and strong flash DA 10 full-field ERG waveforms are profoundly electronegative, (a-waves larger than b-waves) with a-wave amplitudes at the lower reference limit. Oscillatory potential op2 shows most attenuation, but OPs are relatively well preserved. LA 3 and 30Hz flicker ERGs peak times within reference range but amplitudes are at the lower reference limit.

**Supplemental Figure S3**


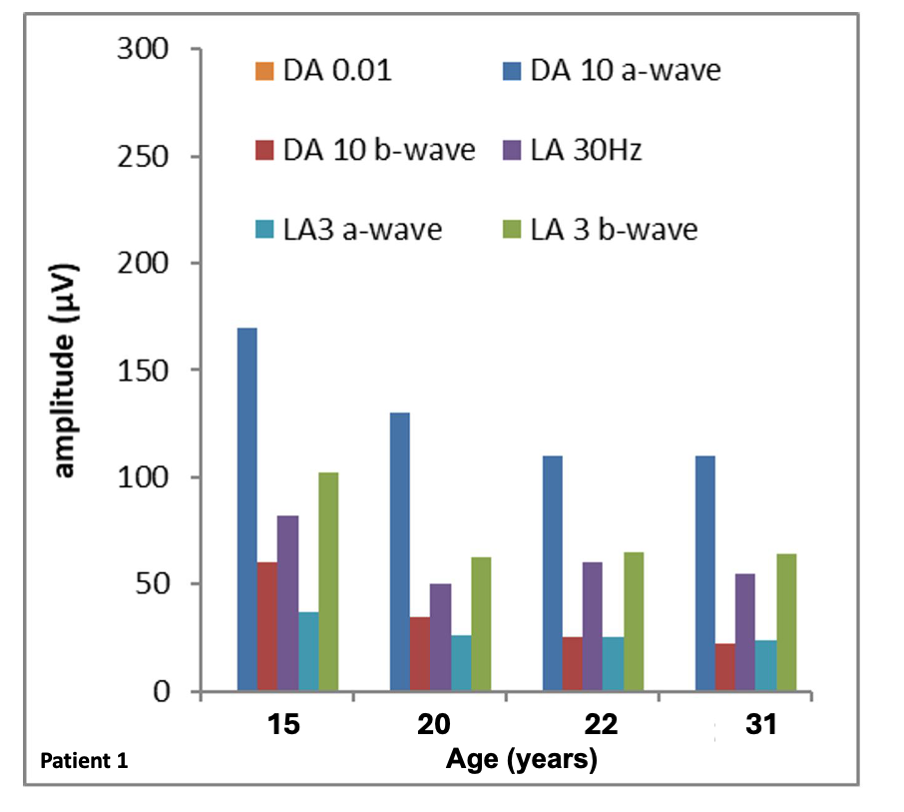


Graphs showing ISCEV standard DA and LA ERG component amplitudes measured on four occasions for patient 1 over a 16-year period. There was mild progressive reduction of DA and LA ERGs, worst between ages 15 and 20 years. LA 30Hz peak times were normal initially (25ms) but worsened to 28ms by age 20 years and to 30ms subsequently (ages 22 and 31 years), consistent with mild worsening of cone system function.

**2. Biochemical testing**

**Methods**

Sulfatase enzyme activities were assessed in plasma and leukocytes, and the urinary excretion of glycosaminoglycan and sulfatides was also evaluated.

Enzyme assays were conducted to measure enzyme activity by quantifying the rate of the reaction. The substrates employed in these tests included synthetic analogues, 4-methylumbelliferyl and p-nitrocatechol sulfate tagged with a fluorescent label. Enzymatic activity was assessed based on the fluorescence released as a result of the enzyme’s interaction with these substrates.

**Supplemental Table S1 Enzymatic Activity Results**

Lab-specific normal reference ranges are shown in brackets below actual values.

Abbreviations: ptn, protein; ↓ , reduced ; ↓↓, significantly reduced

**3. Genetic testing**

All three patients were investigated through a robust clinical pipeline including whole genome sequencing (WGS) followed by bioinformatic analysis focused on candidate variants within a virtual gene panel [Genomics England PanelApp (15); available at <https://panelapp.genomicsengland.co.uk/>] tailored to the patient’s clinical phenotype.

Following WGS, patients 1 and 2 were initially analysed using the Panelapp “posterior segment abnormalities” virtual gene panel, which excluded pathogenic genotypes in known inherited retinal disease (IRD) genes. Patient 1 was analysed using panel v2.6, covering 201 genes, and patient 2 with panel v1.86, covering 177 genes.

Patient 3 underwent analysis using the “undiagnosed metabolic disorders” Panelapp gene panel (v1.78, encompassing 544 genes, including *SUMF1*).

**Supplemental references**

1. Robson AG, Frishman LJ, Grigg J, Hamilton R, Jeffrey BG, Kondo M, et al. ISCEV Standard for full-field clinical electroretinography (2022 update). Doc Ophthalmol. 2022 Jun;144(3):165–77.

2. Thompson DA, Bach M, McAnany JJ, Šuštar Habjan M, Viswanathan S, Robson AG. ISCEV standard for clinical pattern electroretinography (2024 update). Doc Ophthalmol. 2024 Apr;148(2):75–85.

3. Thompson DA, Fujinami K, Perlman I, Hamilton R, Robson AG. ISCEV extended protocol for the dark-adapted red flash ERG. Doc Ophthalmol. 2018 Jun;136(3):191–7.

4. Robson AG, Nilsson J, Li S, Jalali S, Fulton AB, Tormene AP, et al. ISCEV guide to visual electrodiagnostic procedures. Doc Ophthalmol. 2018 Feb;136(1):1–26.

5. Marmoy OR, Moinuddin M, Thompson DA. An alternative electroretinography protocol for children: a study of diagnostic agreement and accuracy relative to ISCEV standard electroretinograms. Acta Ophthalmol. 2022 May;100(3):322–30.

6. Thompson DA, Lyons RJ, Russell-Eggitt I, Liasis A, Jägle H, Grünewald S. Retinal characteristics of the congenital disorder of glycosylation PMM2-CDG. J Inherit Metab Dis. 2013 Nov;36(6):1039–47.

7. Thompson DA, Lyons RJ, Liasis A, Russell-Eggitt I, Jägle H, Grünewald S. Retinal on-pathway deficit in congenital disorder of glycosylation due to phosphomannomutase deficiency. Arch Ophthalmol. 2012 Jun;130(6):712–9.

8. Tzetzi D, Hamilton R, Robinson PH, Dutton GN. Negative ERGs in mucopolysaccharidoses (MPS) Hurler-Scheie (I-H/S) and Hurler (I-H)-syndromes. Doc Ophthalmol. 2007 May;114(3):153–8.

9. Weleber RG. The dystrophic retina in multisystem disorders: the electroretinogram in neuronal ceroid lipofuscinoses. Eye (Lond). 1998;12 ( Pt 3b):580–90.

10. McBain VA, Egan CA, Pieris SJ, Supramaniam G, Webster AR, Bird AC, et al. Functional observations in vitamin A deficiency: diagnosis and time course of recovery. Eye (Lond). 2007 Mar;21(3):367–76.

11. Mukhopadhyay R, Sergouniotis PI, Mackay DS, Day AC, Wright G, Devery S, et al. A detailed phenotypic assessment of individuals affected by MFRP-related oculopathy. Mol Vis. 2010 Mar 26;16:540–8.

12. Sergouniotis PI, Sohn EH, Li Z, McBain VA, Wright GA, Moore AT, et al. Phenotypic variability in RDH5 retinopathy (Fundus Albipunctatus). Ophthalmology. 2011 Aug;118(8):1661–70.

13. Zeitz C, Robson AG, Audo I. Congenital stationary night blindness: an analysis and update of genotype-phenotype correlations and pathogenic mechanisms. Prog Retin Eye Res. 2015 Mar;45:58–110.

14. Ba-Abbad R, Holder GE, Robson AG, Neveu MM, Waseem N, Arno G, et al. Isolated rod dysfunction associated with a novel genotype of CNGB1. Am J Ophthalmol Case Rep. 2019 Jun;14:83–6.

15. Martin AR, Williams E, Foulger RE, Leigh S, Daugherty LC, Niblock O, et al. PanelApp crowdsources expert knowledge to establish consensus diagnostic gene panels. Nat Genet. 2019 Nov;51(11):1560–5.
